# Comparative transcriptome analysis of rainbow trout gonadal cells (RTG-2) infected with U and J genogroup infectious hematopoietic necrosis virus

**DOI:** 10.3389/fmicb.2022.1109606

**Published:** 2023-01-17

**Authors:** Jing-Zhuang Zhao, Li-Ming Xu, Guang-Ming Ren, Yi-Zhi Shao, Qi Liu, Chun-Bo Teng, Tong-Yan Lu

**Affiliations:** ^1^Cell Biology Laboratory, College of Life Science, Northeast Forestry University, Harbin, China; ^2^Heilongjiang River Fisheries Research Institute, Chinese Academy of Fishery Sciences, Harbin, China; ^3^Key Laboratory of Aquatic Animal Diseases and Immune Technology of Heilongjiang Province, Harbin, China

**Keywords:** rainbow trout gonadal cells, infectious hematopoietic necrosis virus, transcriptome analysis (RNAseq), U genogroup, J genogroup

## Abstract

Infectious hematopoietic necrosis virus (IHNV) is the causative pathogen of infectious hematopoietic necrosis, outbreaks of which are responsible for significant losses in rainbow trout aquaculture. Strains of IHNV isolated worldwide have been classified into five major genogroups, J, E, L, M, and U. To date, comparative transcriptomic analysis has only been conducted individually for the J and M genogroups. In this study, we compared the transcriptome profiles in U genogroup and J genogroup IHNV-infected RTG-2 cells with mock-infected RTG-2 cells. The RNA-seq results revealed 17,064 new genes, of which 7,390 genes were functionally annotated. Differentially expressed gene (DEG) analysis between U and J IHNV-infected cells revealed 2,238 DEGs, including 1,011 downregulated genes and 1,227 upregulated genes. Among the 2,238 DEGs, 345 new genes were discovered. The DEGs related to immune responses, cellular signal transduction, and viral diseases were further analyzed. RT-qPCR validation confirmed that the changes in expression of the immune response-related genes *trpm2*, *sting*, *itgb7*, *ripk2*, and *irf1*, cellular signal transduction-related genes *irl*, *cacnb2*, *bmp2l*, *gadd45α*, and *plk2*, and viral disease-related genes *mlf1, mtor, armc5*, *pik3r1*, and *c-myc* were consistent with the results of transcriptome analysis. Taken together, our findings provide a comprehensive transcriptional analysis of the differential virulence of the U and J genogroups of IHNV, and shed new light on the pathogenic mechanisms of IHNV strains.

## 1. Introduction

Rainbow trout is an important freshwater fish species in Chinese aquaculture that is valued for its nutrient-rich meat. However, rainbow trout farming is severely affected worldwide by viral diseases (MacWilliams et al., 2007; Ji et al., 2017; Zhao et al., 2017). One of the most severe viral diseases is infectious hematopoietic necrosis (IHN), which has caused significant losses in rainbow trout aquaculture since it was first reported in China (Niu and Zhao, 1988).

IHN disease is caused by infectious hematopoietic necrosis virus (IHNV) that contains a non-segmented single-stranded, negative-sense RNA and belongs to the genus *Novirhabdovirus* in the family *Rhabdoviridae* (Wargo et al., 2017). The viral genome is approximately 11 kb in length and contains five structural proteins and a non-virion protein (Kurath et al., 2003). Phylogenetic analyses have classified IHNV strains, isolated worldwide, into five major genogroups: J, E, L, M, and U (Xu et al., 2019). The genogroups U, M, and L were first detected in North America (Kurath et al., 2003; Black et al., 2016), and the E genogroup is prevalent in Europe (Enzmann et al., 2005; Cieslak et al., 2017). The first reported outbreak of IHN in Asia was from Japanese salmonid farming factories and it was associated with importation of sockeye salmon eggs from Alaska (Kimura and Yoshimizu, 1991; Nishizawa et al., 2006). Genetic typing analysis showed that the original IHNV in Japan was sockeye-specific U genogroup, but it jumped into rainbow trout as the virus spread in Japan (Nishizawa et al., 2006). During the 1980s, U genogroup evolved into J genogroup and became virulent in rainbow trout (Garver et al., 2006). Our study, along with previously reported studies, confirmed that all IHNV isolates from China belong to the J genogroup highly virulent in rainbow trout (Xu et al., 2019).

A few previous reports have detailed the pathogenic differences between J and U genogroup IHNV infection (Park et al., 2011; Xu et al., 2017), but the molecular mechanisms that underlie the pathogenic differences between these genogroups remain poorly understood. High-throughput sequencing technology, which is widely used in pathogenic and immune-related studies, permits genome wide transcriptomic analysis at high resolution. In the present study, we present the transcriptional sequencing libraries of U or J genogroup virus-infected, or PBS mock-infected rainbow trout gonadal (RTG-2) cells at 24 h post-infection. The different gene expression profiles were analyzed and identified by comparisons between the transcriptional sequencing libraries, and were then validated by RT-qPCR. A great number of genes that were differentially expressed upon J and U genogroup IHNV infection were identified and functionally annotated. This is the first report of transcriptional analysis between the U and J genogroups of IHNV. Our findings not only provide comprehensive transcriptional analysis, but also identify possible mechanisms associated with the differential virulence of the U and J genogroups of IHNV.

## 2. Materials and methods

### 2.1. Virus strains and cells

The RTG-2 cell line (CCL-55, ATCC) was cultured in Eagle’s minimum essential medium (MEM, 8118228, Gibco, Suzhou, Jiangsu, China) with 10% fetal bovine serum (FBS) and 0.1 mg/ml streptomycin and penicillin. The U genogroup IHNV strain Blk94 (GenBank no: DQ164100) was kindly provided as a gift by Dr. Hong Liu of the Shenzhen Entry-exit Inspection and Quarantine Bureau, China. The J genogroup IHNV strain Sn1203 (GenBank no: KC660147.1) was isolated from moribund rainbow trout fry in China and was stored in our laboratory.

### 2.2. IHNV infection and samples collection

RTG-2 cells were infected with either IHNV virus (U or J genogroup IHNV) at a multiplicity of infection of 1.0, or mock infected with phosphate-buffered saline (PBS, control). After 1 h incubation, the treated RTG-2 cells were washed three times with PBS and then cultured in MEM with 2% FBS at 15°C. At 24 h post-IHNV-infection, the cells were collected and used for further analysis.

### 2.3. RNA extraction, and mRNA library construction and sequencing

Total RNA was extracted from RTG-2 cells treated with different IHNV strains using TRIzol reagent (15,596,026, Invitrogen, Carlsbad, California, United States) according to the manufacturer’s instructions, and then digested with DNase I. The RNA integrity was determined using an Agilent 2,100 Bioanalyzer (Agilent Technologies, California, United States). The RNA samples that had a 28S/18S rRNA ratio of greater than 1.9 and an RNA integrity ≥8 were chosen for library construction (three individual samples per treatment group). The mRNA was isolated and fragmented from 1 μg of total RNA, followed by double-stranded cDNA synthesis. The 3′ end was repaired and adenylated for adapter ligation and finally, reverse PCR amplification. The final libraries for sequencing were analyzed using an Agilent 2,100 Bioanalyzer and quantified by qPCR using a Library Quantification Kit Illumina (KK4602, Kapa, Boston, Massachusetts, United States) according to the manufacturer’s instructions.

### 2.4. Data processing and analysis of differential gene expression

Raw data were filtered by removing adaptor sequences and low quality sequences using SOAPnuke (v1.5.2) to obtain high quality clean data. The clean data were then mapped to the *Oncorhynchus mykiss* genome sequence^1^ using a HISAT2 comparison system (Kim et al., 2015). The mapped reads were assembled and quantified by StringTie comparisons (Pertea et al., 2015), and high quality data were used for downstream analysis. The mapped data were then annotated by BLAST against the NR, Swiss-Prot, COG, KOG, KEGG, GO, Pfam, eggNOG, and TrEMBL databases. Fragments Per Kilobase of transcript per Million fragments mapped (FPKM) served as a measured standard of the transcript or gene expression level. The screening criteria to identify significantly differentially expressed genes (DEGs) were a fold-change ≥2 and a false discovery rate (FDR) < 0.01. The DESeq2 software was used for DEG analysis involving three comparisons (Mock vs. U, Mock vs. J, and U vs. J).

### 2.5. RT-qPCR analysis of gene expression

To validate the results of RNA-seq analysis, 15 of the DEGs between the U and J groups relating to immune responses, cellular signal transduction, and viral diseases, were analyzed by RT-qPCR. The total RNA used for cDNA library construction was used for RT-qPCR analysis. The total RNA was first transcribed to cDNA using a PrimeScript RT Reagent Kit with gDNA Eraser (RR047A, Takara, Japan), and then a qPCR assay was performed using a TB Green Premix Ex Taq II Kit (RR820A, Takara). The primers used for RT-qPCR are listed in Supplementary Table 1, and β-actin was used as an internal control. The relative expression levels of different genes were calculated using the 2^˗ΔΔCT^ method.

### 2.6. Statistical analysis

The data were analyzed using the GraphPad Prism software (version 8.0) and are presented as the mean ± standard deviation (SD). The differences between groups were analyzed by a Student’s *t*-test, and a statistically significant difference was defined as a *value of p* < 0.05.

## 3. Results

### 3.1. Quality analysis of transcriptome sequencing

In this study, nine cDNA libraries were obtained from RTG-2 cells after J-and U-IHNV infection. The quality control results showed that 63,513,756–66,846,372 raw reads and 60,573,286–63,305,380 clean reads were obtained from the nine cDNA libraries, and the ratio of clean reads ranged from 93.12 to 95.53%. The Q20 and Q30 values for all of the libraries were over 99.01 and 92.23%, respectively, and indicated the high quality of the RNA sequence data (Table 1). The raw sequence data have been deposited in the NCBI Sequence Read Archive, under accession number PRJNA895736. The obtained clean reads were mapped to the *O. mykiss* genome sequence and the mapping rates ranged from 88.58 to 93.71%, with most reads being mapped to exon regions within the *O. mykiss* genome.

**Table 1:** Summary of the quality analysis of RNA-seq data.

Sample name	Raw reads	Clean reads	Ratio of clean reads (%)	% ≥ Q20	% ≥ Q30	Mapped reads	Mapped rate (%)
Mock01	63,513,756	60,573,286	95.37	99.18	92.77	53,658,025	88.58
Mock02	65,146,218	61,991,656	95.16	99.06	92.23	54,945,172	88.63
Mock03	66,133,174	63,045,716	95.33	99.17	93.46	56,146,882	89.06
J24h01	66,352,932	61,787,254	93.12	99.45	93.86	57,719,634	93.42
J24h02	66,846,372	62,891,688	94.08	99.30	93.76	58,844,980	93.57
J24h03	64,442,328	61,562,360	95.53	99.45	93.87	57,690,477	93.71
U24h01	66,821,192	63,305,380	94.74	99.28	93.62	56,760,424	89.66
U24h02	66,062,330	62,792,522	95.05	99.25	93.52	56,232,737	89.56
U24h03	66,244,510	61,773,066	93.25	99.01	93.28	55,215,857	89.39

### 3.2. Functional annotation and classification

The RNA-seq analysis revealed 17,064 new genes, 7,390 of which were functionally annotated. To understand the functions of these 7,390 genes, nine databases were used to annotate the unigenes. Of the 7,390 genes, the following numbers of genes were annotated using the following databases: 294 (3.98%) in COG, 3,729 (50.46%) in GO, 3,699 (50.05%) in KEGG, 1,320 (17.86%) in KOG, 2,248 (30.42%) in Pfam, 710 (9.61%) in Swiss-Prot, 7,053 (95.44) in TrEMBL, 3,701 (50.08%) in eggNOG, and 6,773 (91.65%) in Nr (Supplementary Table 2).

**Figure 1:**
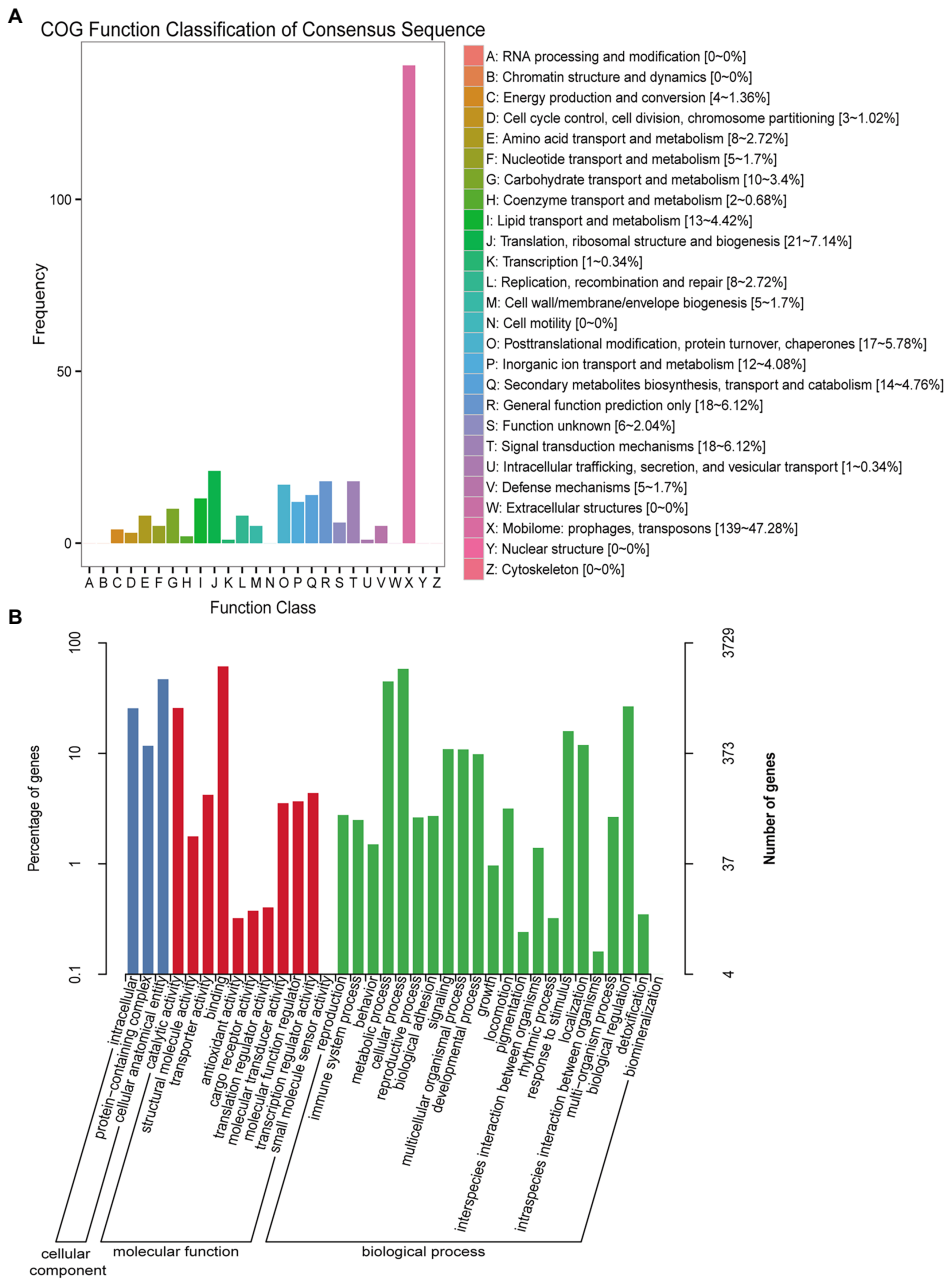
COG and GO function classification of unigenes from U-and J-IHNV-infected cells. **(A)** COG function classification of unigenes. **(B)** GO function classification of unigenes.

Among the COG functional annotations, 294 unigenes were classified into 20 categories, with the largest group being the mobilome: prophages, transposons (139, 47.28%), followed by translation, ribosomal structure and biogenesis (21, 7.14%), general function prediction only (18, 6.12%), and signal transduction mechanisms (18, 6.12%). Among the 25 categories in COG functional annotation, no unigenes were classified in RNA processing and modification, chromatin structure and dynamics, nuclear structure, cell motility, cytoskeleton, and extracellular structures (Figure 1A; Supplementary Table 2).

Among the GO functional annotation, the 3,729 unigenes were classified into 3 cellular components, 11 molecular functions, and 22 biological processes. The predominant category was cellular anatomical entity (1,749, 46.90%) in cellular component, binding (2,287, 61.33%) in molecular functions, and cellular process (2,172, 58.25%) in biological process. Of note, in the category biological process, there were 13 unigenes (0.35%) annotated in detoxification, 93 unigenes (2.49%) annotated in immune system process, and 593 unigenes (15.90%) annotated in response to stimulus (Figure 1B; Supplementary Table 2).

The KEGG pathway analysis revealed that 3,699 unigenes were categorized into 184 KEGG pathways, and the three predominant enriched categories were herpes simplex virus 1 infection (701, 18.95%), calcium signaling pathway (110, 2.97%), and protein processing in endoplasmic reticulum (105, 2.84%; Supplementary Table 2).

### 3.3. Identification of differentially expressed genes (DEGs) between the mock versus J and mock versus U groups

#### 3.3.1. DEG analysis in the mock versus J group

**Figure 2:**
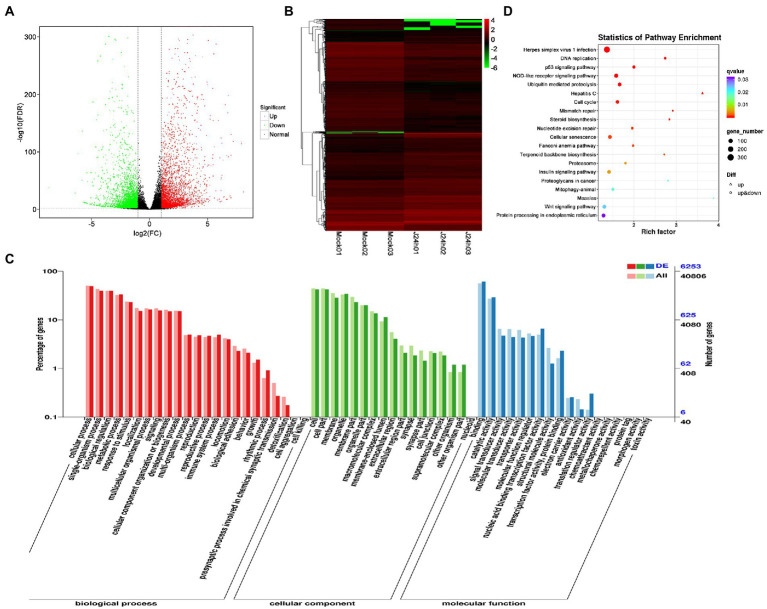
Comparison and enrichment analysis of DEGs in the Mock versus J-IHNV infection groups. **(A)** Volcano plot of DEGs. **(B)** Hierarchical clustering analysis of DEGs. **(C)** GO enrichment of DEGs. **(D)** KEGG enrichment analysis of DEGs.

DEG analysis revealed 8,296 DEGs in the Mock vs. J-IHNV infected group, including 4,058 up-regulated genes and 4,238 down-regulated genes (Figure 2A; Supplementary Table 3). Among the 8,296 DEGs, 1,056 new genes were discovered (Supplementary Table 3). Hierarchical clustering analysis showed that J-IHNV infection significantly changed the host gene expression levels compared with the Mock-infected group (Figure 2B). GO enrichment analysis revealed that 6,253 DEGs were annotated, and the top five enriched categories were binding (3,817, 61.04%), cellular process (3,062, 48.97%), cell (2,657, 42.49%), cell part (2,651, 42.40%), and single-organism process (2,466, 39.44%; Figure 2C; Supplementary Table 3). To better understand the effects of J-IHNV infection on pathway changes, KEGG analysis was performed and 6,000 DEGs were annotated into 248 pathways (Supplementary Table 3). The top 20 significantly enriched pathways included herpes simplex virus 1 infection, DNA replication, p53 signaling pathway, NOD-like receptor signaling pathway, and ubiquitin mediated proteolysis (Figure 2D; Supplementary Table 3).

#### 3.3.2. DEG analysis in the mock versus U group

**Figure 3:**
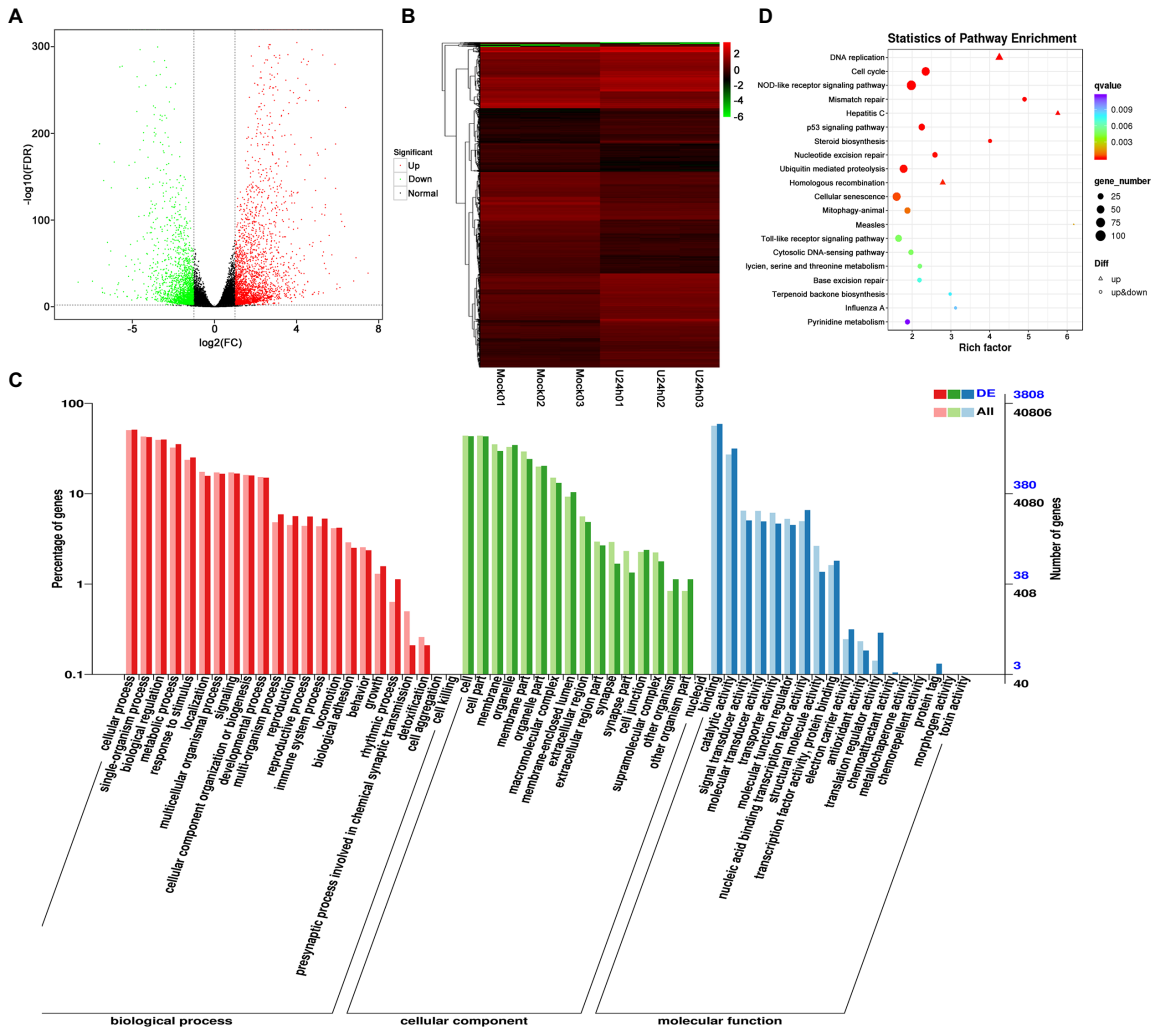
Comparison and enrichment analysis of DEGs in the Mock versus U-IHNV infection groups. **(A)** Volcano plot of DEGs. **(B)** Hierarchical clustering analysis of DEGs. **(C)** GO enrichment of DEGs. **(D)** KEGG enrichment analysis of DEGs.

DEG analysis revealed 5,000 DEGs in the Mock vs. U-IHNV infected group, including 2,725 up-regulated genes and 2,275 down-regulated genes (Figure 3A; Supplementary Table 4). Among the 5,000 DEGs, 653 new genes were discovered (Supplementary Table 4). Hierarchical clustering analysis showed that U-IHNV infection significantly changed the host gene expression level compared with the Mock-infected group (Figure 3B). GO enrichment analysis showed that 3,808 DEGs were annotated and the top five enriched categories were the same as for the J-IHNV infection group (Figure 3C; Supplementary Table 4). KEGG analysis was performed and 3,627 DEGs were annotated into 229 pathways (Supplementary Table 4). The top 20 significantly enriched pathways included DNA replication, cell cycle, NOD-like receptor signaling pathway, mismatch repair, and hepatitis C (Figure 3D; Supplementary Table 4).

### 3.4. DEG analysis in the U versus J group

**Figure 4:**
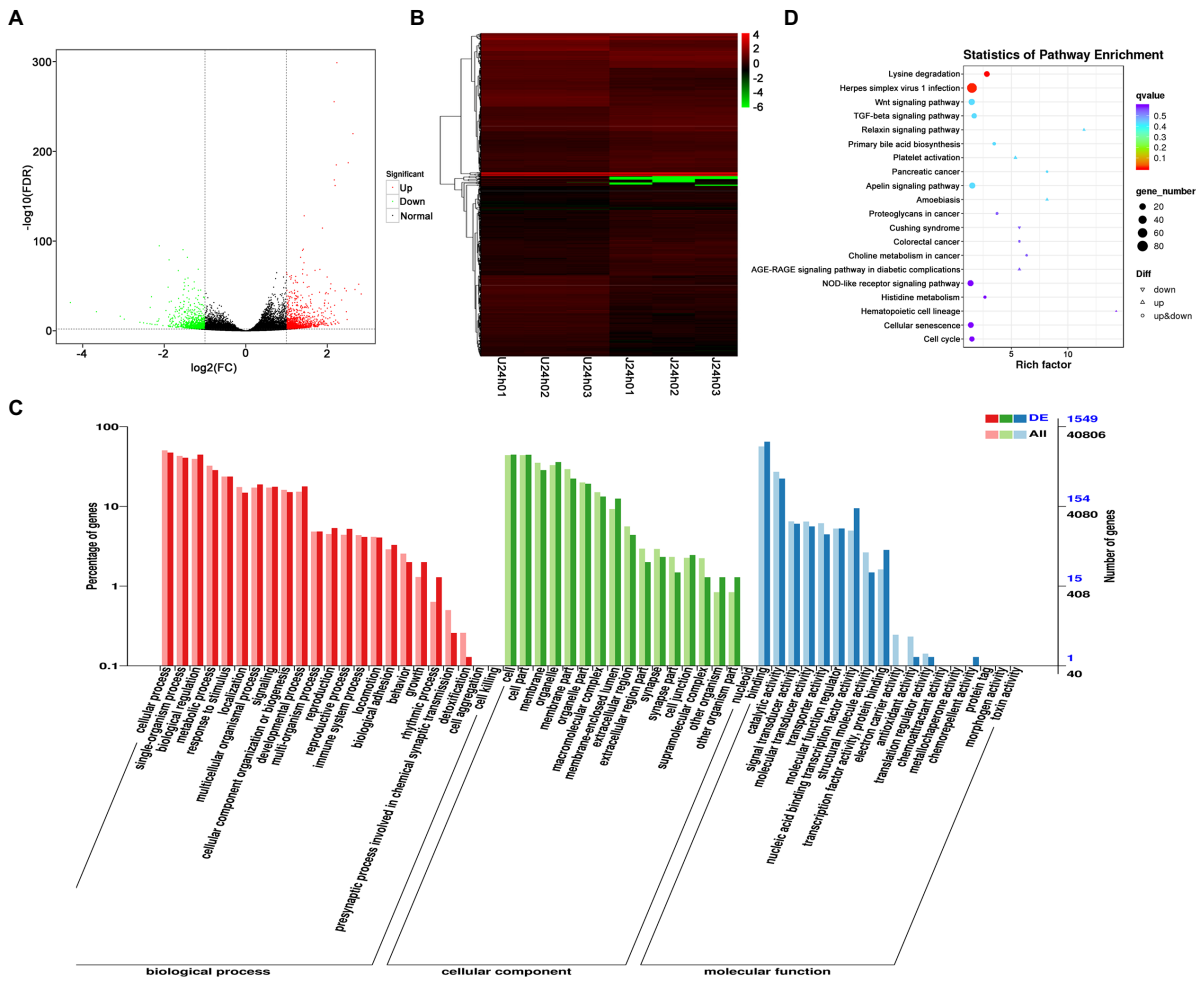
Comparison and enrichment analysis of DEGs in the U versus J-IHNV infection groups. **(A)** Volcano plot of DEGs. **(B)** Hierarchical clustering analysis of DEGs. **(C)** GO enrichment of DEGs. **(D)** KEGG enrichment analysis of DEGs.

DEG analysis revealed 2,238 DEGs in the U vs. J IHNV infected group, including 1,227 up-regulated genes and 1,011 down-regulated genes (Figure 4A; Supplementary Table 5). Among the 2,238 DEGs, 345 new genes were discovered (Supplementary Table 5). Hierarchical clustering analysis showed that host gene expression levels were significantly changed between the U-and J-IHNV infection groups (Figure 4B). GO enrichment analysis revealed that 1,549 DEGs were annotated and the top five enriched categories were binding, cellular process, biological regulation, cell, and cell part (Figure 4C; Supplementary Table 5). KEGG analysis was performed and 1,525 DEGs were annotated into 187 pathways (Supplementary Table 5). The top 20 significantly enriched pathways included lysine degradation, herpes simplex virus 1 infection, relaxin signaling pathway, primary bile acid biosynthesis, platelet activation, and TGF-beta signaling pathway (Figure 4D; Supplementary Table 5).

#### 3.4.1. Analysis of DEGs related to immune responses

**Figure 5:**
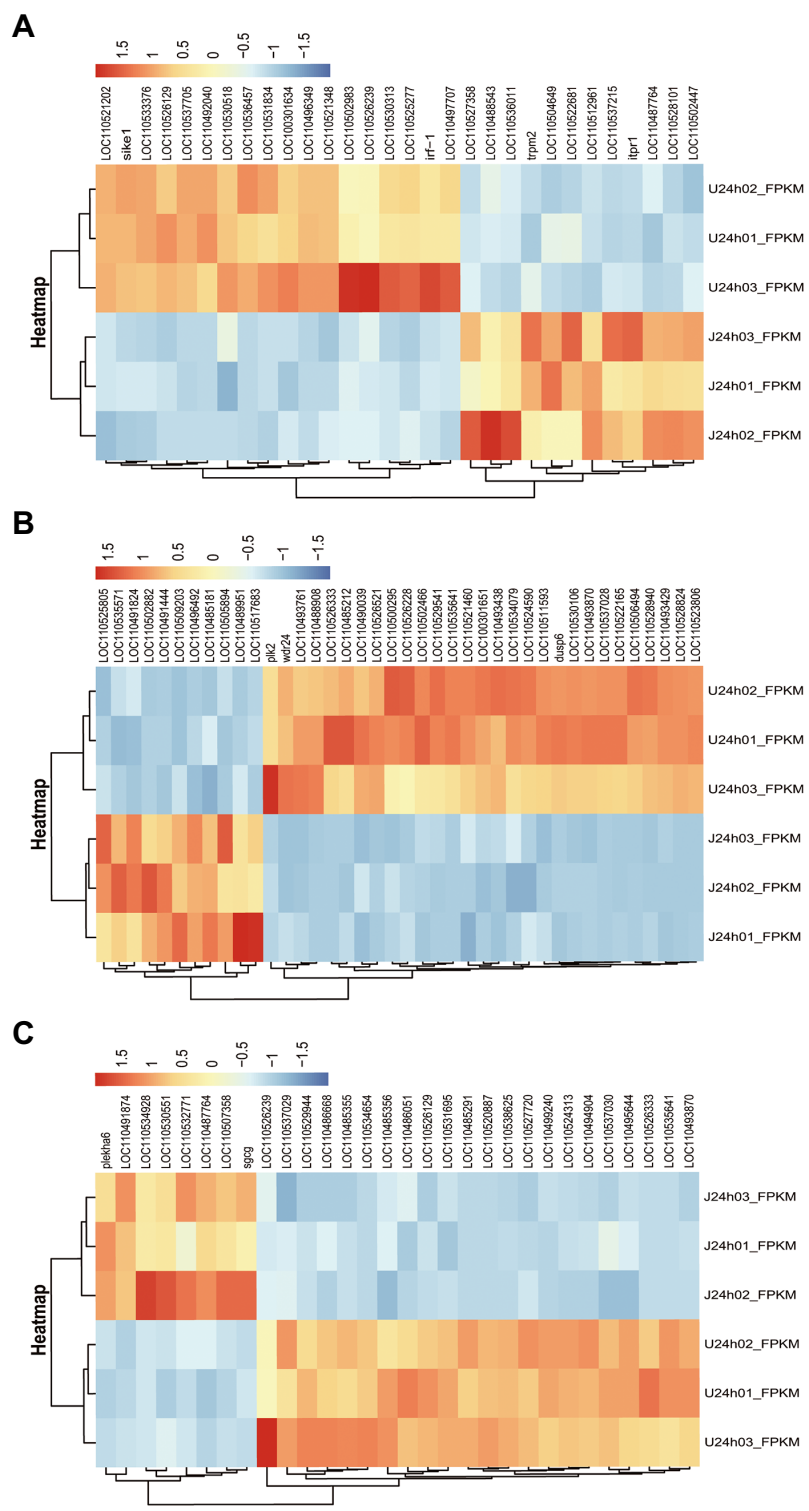
DEG analysis of the U versus J-IHNV infection groups for genes related to immune responses, cellular signal transduction, and viral diseases. **(A)** Top 30 DEGs related to immune responses. **(B)** Top 40 DEGs related to cellular signal transduction. **(C)** Top 30 DEGs related to viral diseases.

The results showed that DEGs between U-and J-IHNV mapped to 187 KEGG pathways, and 15 of these pathways were significantly enriched (*p* < 0.05; Supplementary Table 5). Of particular interest, the DEGs between the U-and J-IHNV infection groups were enriched in immune-related pathways, such as the C-type lectin receptor signaling pathway, NOD-like receptor signaling pathway, RIG-I-like receptor signaling pathway, and Toll-like receptor signaling pathway (Supplementary Table 5). To further understand the DEGs between the U and J-IHNV infection groups, the top 30 DEGs related to immune responses were screened from the 187 KEGG pathways (Figure 5A). The results showed that J-IHNV infection increased the expression of the transient receptor potential cation channel, subfamily M, member 2 (TRPM2) by 1.46-fold, stimulator of interferon genes protein-like (STING, LOC110487764) by 1.45-fold, integrin beta-7 (ITGB7, LOC110528101) by 1.20-fold, SLIT and NTRK-like protein 4 (SLITRK4, LOC110488543) by 1.12-fold, and anthrax toxin receptor 1 (ANTXR1, LOC110537215) by 1.11-fold compared with expression levels following U-IHNV infection. By contrast, the expression levels of anthrax toxin receptor 2 (ANTXR2, LOC110536457), arrestin domain-containing protein 2 (ARRDC2, LOC110492040), receptor-interacting serine/threonine-protein kinase 2 (RIPK2, LOC110526239), thioredoxin-interacting protein (TXNIP, LOC110496349), and interferon regulatory factor 1 (IRF1, LOC110533376) in the J-IHNV infection group were decreased by 2.42-fold, 2.17-fold, 2.17-fold, 2.12-fold, and 1.86-fold, respectively, compared with the U-IHNV infection group (the fold change was represented as log_2_^FC^; Supplementary Table 6).

#### 3.4.2. Analysis of DEGs related to cellular signal transduction

The DEGs between the U and J IHNV infection groups were also enriched in signal transduction related pathways, such as the MAPK signaling pathway, Wnt signaling pathway, calcium signaling pathway, neuroactive ligand-receptor interaction pathway, and apelin signaling pathway (Supplementary Table 5). The top 40 DEGs related to signal transduction were screened to further understand the differences in signal transduction between the U and J IHNV infection groups (Figure 5B). The results showed that J-IHNV infection increased the expression of casein kinase I (CK1, LOC110502882) by 1.90-fold, insulin receptor-like (IRL, LOC110509203) by 1.84-fold, calcium channel voltage-dependent beta 2a (CACNB2, LOC110489951) by 1.74-fold, bone morphogenetic protein 2-like (BMP2L, LOC110491444) by 1.60-fold, and E3 ubiquitin-protein ligase RNF152 (RNF152, LOC110535571) by 1.56-fold compared with the expression levels following U-IHNV infection. By contrast, the expression levels of dual specificity protein phosphatase 4 (DUSP4, LOC110523806), transcription factor 7-like 1-A (TCF7L1A, LOC110526333), growth arrest and DNA damage-inducible protein GADD45 alpha (Gadd45α, LOC110530106), serine/threonine-protein kinase PLK2 (PLK2), and cellular communication network factor 2a (CCN2, LOC110522165) in the J-IHNV infection group were decreased by 2.31-fold, 1.86-fold, 1.85-fold, 1.81-fold, and 1.66-fold, respectively, compared with the U-IHNV infection group (the fold change was represented as log_2_^FC^; Supplementary Table 6).

#### 3.4.3. Analysis of DEGs related to viral diseases

The DEGs between the U and J IHNV infection groups were also enriched in viral disease-related genes, such as herpes simplex virus 1 infection, salmonella infection, and endocrine and metabolic disease (Supplementary Table 5). The top 30 DEGs related to disease were screened and analyzed (Figure 5C). The results showed that J-IHNV infection increased the expression of pleckstrin homology domain-containing family A member 6 (PLEKHA6) by 1.53-fold, tumor necrosis factor receptor superfamily member 14-like (TNFRSF14L, LOC110532771) by 1.19-fold, myeloid leukemia factor 1 (MLF1, LOC110507358) by 1.50-fold, myomesin-3-like (MYOM3L, LOC110534928) by 1.15-fold, and serine/threonine-protein kinase mTOR (LOC110491874) by 1.31-fold compared with the expression levels following U-IHNV infection. By contrast, the expression levels of CDC42 effector protein (Rho GTPase binding) 4b (CDC42EP4b, LOC110499240), armadillo repeat-containing protein 5 (ARMC5, LOC110538625), protein S100-A1 (S100-A1, LOC110520887), phosphatidylinositol 3-kinase regulatory subunit alpha (PIK3R1, LOC110526129), and transcriptional regulator Myc-like (c-Myc, LOC110535641) in the J-IHNV infection group were decreased by 1.32-fold, 2.14-fold, 1.89-fold, 1.33-fold, and 1.51-fold, compared with the U-IHNV infection group (the fold change was represented as log_2_^FC^; Supplementary Table 6).

### 3.5. DEG validation by RT-qPCR

**Figure 6:**
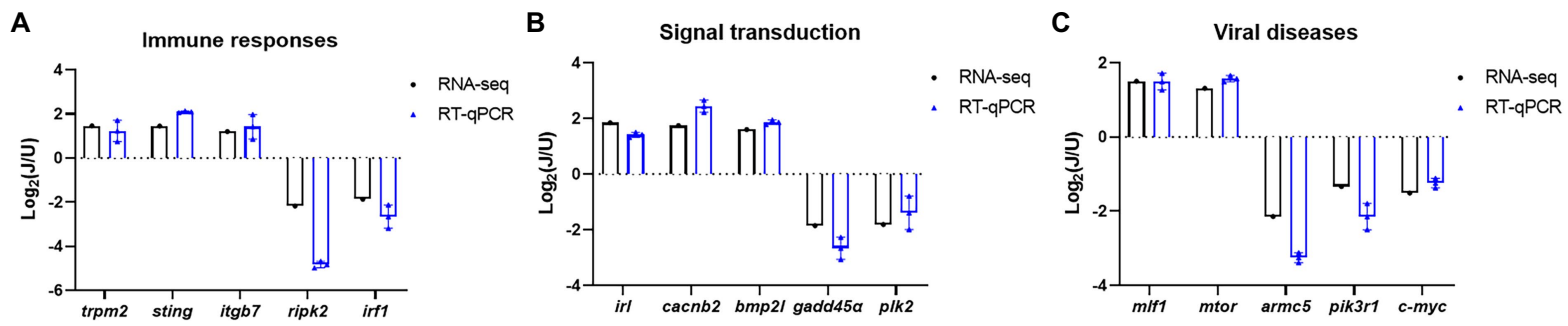
Validation of selected DEGs related to immune responses, cellular signal transduction, and viral diseases. **(A)** DEG expression levels for genes related to immune responses. **(B)** DEG expression levels for genes related to cellular signal transduction. **(C)** DEG expression levels for genes related to viral diseases.

To validate the results of RNA-seq analysis, some of the DEGs between the U and J IHNV infection groups related to the immune response, signal transduction, and disease were analyzed by RT-qPCR. The results showed that immune response related genes *trpm2*, *sting*, and *itgb7*, signal transduction-related genes *irl*, *cacnb2*, and *bmp2l*, and disease-related genes *mlf1* and *mtor* were all up regulated, and immune response-related genes *ripk2* and *irf1*, signal transduction-related genes *gadd45α* and *plk2*, and disease-related genes *armc5*, *pik3r1* and *c-myc* were all down regulated in the J-IHNV group compared with the U-IHNV group (Figure 6). The fold-changes in DEGs determined by RT-qPCR were consistent with the changes derived from RNA-seq analysis, confirming the reliability of the RNA-seq data.

## 4. Discussion

Rainbow trout is an important cultured fish worldwide that significantly contributes to the economy of the fishery industry, but viral disease outbreaks severely reduce fish production. One of the most severe viral diseases of rainbow trout is caused by IHNV, which can result in a mortality rate of more than 90% (Zhao et al., 2019). Phylogenetic analyses classified IHNV strains, which have been isolated worldwide, into five major genogroups (Xu et al., 2019). IHNV isolates from China belong to the J genogroup, which has evolved from the U genogroup (Garver et al., 2006). The J genogroup virus is highly virulent in rainbow trout, whereas the U genogroup virus is lentogenic. Although some articles have investigated the pathogenicity of J and U genogroup IHNV, the molecular mechanisms that underlie the pathogenic differences remain poorly understood. To study the pathogenic mechanisms of these two viruses in detail, we conducted genome wide transcriptomic analysis at high resolution to assess viral-caused changes in immune responses, cellular signal transduction, and viral disease-related genes.

Host immune responses provide a critical biological barrier to protect the host against pathogen invasion, and many immune mechanisms are conserved among vertebrates. Given the crucial role of the immune response, immune system-related DEGs were further analyzed after U-and J-IHNV infection. The results showed that *trpm2*, *sting*, and *itgb7* in J-IHNV-infected cells were significantly up-regulated compared with U-IHNV-infected cells, and the *ripk2* and *irf1* genes were significantly down-regulated (Figure 6). TRPM2 is a reactive oxygen species-sensitive protein that is widely distributed and played an important role in the immune system (Xia et al., 2019). It was previously reported that HBV infection promotes TRPM2 expression to facilitate virus replication, while the inhibition of TRPM2 protects cells from H9N2 virus-induced damage (Wang et al., 2020; Chen et al., 2022). ITGB7 is a protein that associates with ITGA4 to form LPAM-1 dimer that functions as a T cell and B cell adhesion receptor and is important for intestinal immunity (Ballet et al., 2021). A study on Epstein–Barr virus (EBV) showed that virus infection induced the expression of ITGB7, which was beneficial for EBV infection (Delecluse et al., 2019). These results indicated that the TRPM2 and ITGB7 proteins may facilitate virus replication, and suggested that the induced high-level expression of TRPM2 and ITGB7 may facilitate higher replicative ability and pathogenicity of J-IHNV. STING is an important innate immune response protein that is reported to exert antiviral effects through numerous DNA and RNA-sensing pattern recognition receptors, and loss of STING caused a reduction in IFN production (Ma and Damania, 2016; Bao et al., 2021). STING mediated an IFN response to DNA virus and restricted RNA viruses (such as adenovirus, poxvirus, and vesicular stomatitis virus) by mediating translation inhibition (Lam and Falck-Pedersen, 2014; Franz et al., 2018; Georgana et al., 2018; Lee et al., 2019). Considering that J-IHNV is more virulent than U-IHNV, it may invoke a stronger immune response, including an increase in STING protein, to inhibit pathogen invasion. Although virus invasion results in an enhanced immune response, viruses have evolved in many ways to evade immunity, such as inhibiting the expression levels of some immune-related genes. RIPK2, also known as RIP2, is a protein used by NOD1 and NOD2 to direct the innate immune response against viral and bacterial infections (Hofmann et al., 2021). Research on influenza A virus showed that knockout of RIPK2 caused increased mortality after virus infection and indicated that RIPK2 protects the host against severe influenza A virus infection (Lupfer et al., 2013). IRF1 is a member of the interferon regulatory factor family, which binds to and activates type I interferon gene promoters (Feng et al., 2021). In recent years, IRF1 has been shown to mediate inducible and constitutive host defenses against virus infection, such as Sendai virus (Motz et al., 2013), dengue virus (Odendall et al., 2014), and hepatitis A virus (Yamane et al., 2019). In this study, we found that the expression levels of *ripk2* and *irf1* were downregulated in J-IHNV infection group, indicating that this virus has a stronger inhibitory effect on the immune system than U-IHNV.

Cells respond to a variety of extracellular changes by activating intracellular signal transduction pathways (Ludwig et al., 2021). Viruses are regarded as the extracellular stimuli that can lead to activated signal transduction. The successful activation of signal transduction facilitates various cellular responses against infection and is an important part of the host antiviral response. However, viruses have evolved many mechanisms to misuse or suppress antiviral signal transduction to promote their own replication. Therefore, we analyzed the signal transduction pathway-related DEGs after U and J genogroup IHNV infection. The results showed that the *ck1* and *RNF152* genes were significantly upregulated in J genogroup IHNV-infected cells compared with U IHNV-infected cells and the *dusp4*, *gadd45α*, *plk2*, and *ccn2* genes were significantly down-regulated (Figure 6). The CK1 family is a Ser/Thr protein kinase family reported to serve as regulators in the signal transduction of eukaryotic cells, as well as playing a role in many biological processes, such as regulating the Wnt signal, nucleus-cytoplasm shuttling of transcriptional factors, and DNA repair (Wang et al., 2014). During influenza A virus infection, viral HA protein induced IFN receptor degradation *via* CK1 to promote virus replication (Xia et al., 2018). CK1 regulated barley yellow striate mosaic virus-induced liquid–liquid phase separation and inhibited virus replication (Fang et al., 2022). These findings indicated that CK1 plays different roles in virus infection, and its role may vary from virus to virus. Although *ck1* gene expression in J-IHNV-infected cells was significantly upregulated compared with U-IHNV-infected cells, the exact role of *ck1* during IHNV infection remains to be clarified. RNF152 was reported as a positive regulator of TLR/IL-1R-mediated signaling and potentiates LPS-and IL-1β-induced activation of NF-κB in a ubiquitination-independent manner (Xiong et al., 2020). DUSPs are negative regulators of MAPK signal pathways, and DUSP1 and DUSP4 are believed to have similar specificities for the dephosphorylation of protein (Lin et al., 2020). Research showed that EBV infection significantly down-regulated DUSPs, including DUSP1 and DUSP4 (Lin et al., 2020). DUSP1 silencing increased the IFN antiviral effect against hepatitis C virus (HCV) by increasing the phosphorylation and nuclear translocation of STAT1 (Choi et al., 2015). Gadd45 proteins function as stress sensors in response to various environmental and physiological stressors, including bacterial and viral infection (Schmitz, 2013). HCV infections inhibited the mRNA transcription and protein expression levels of Gadd45 (Chen et al., 2016). PLK2 was a cell cycle regulator and also an apoptotic effector (Liu et al., 2015). Avian metapneumovirus subtype C (aMPV/C) infection significantly upregulated PLK2 expression and contributed to p53-mediated apoptosis to promote virus replication in Vero cells (Quan et al., 2020). However, in some cases, the inhibition of apoptosis may increase the viral titer by increasing the survival of infected cells. CCN2 is a matrix protein that can enhance immune cell migration and proinflammatory cytokine production to facilitate inflammatory responses (Kubota and Takigawa, 2015). Porcine reproductive and respiratory syndrome virus (PRRSV) infection inhibited the mRNA transcription of CCN2 to facilitate virus replication (Park and Chun, 2020). Collectively, compared with U-IHNV, J-IHNV infection caused down-regulation of the *dusp4*, *gadd45α*, *plk2*, and *ccn2* genes in a similar manner to EBV, HCV, aMPV/C, and PRRSV, suggesting that these genes may perform the same role during different virus infections, but the exact role within IHNV remains to be clarified.

In certain cases, virus infection can result in cancer, immune deficiency, drug resistance, endocrine and metabolic disorder, or even death. IHNV infection causes clinical signs in fish that include a darkened body, pale gills, exophthalmos, and hematopoietic tissue necrosis (Enzmann et al., 2010). To better understand the difference between J-IHNV and U-IHNV, the disease-related DEGs were analyzed. We found that in J-IHNV infection, the *tnfrsf14* and *mlf1* genes were significantly up-regulated compared with U-IHNV infection (Figure 6). TNFRSF14 is a cell surface molecule belonging to the TNF receptor superfamily that functions as an activator of cell survival genes *via* NF-kB transcription factors (Ware and Sedy, 2011). In recent years, TNFRSF14 was recognized as an alternative entry receptor for herpes simplex virus, and high protein levels facilitated viral replication (Taylor et al., 2007). MLF1 was first identified from a chromosomal rearrangement that caused acute myeloid leukemia and myelodysplastic syndrome, and functioned in the regulation of primitive hematopoietic cell development (Matsumoto et al., 2000). Based on the function of the TNFRSF14 and MLF1 proteins, we supposed that J-IHNV may induced higher protein levels to ensure the survival of the infected host to achieve increased viral titers, but the exact role of TNFRSF14 and MLF1 still needed to be clarified. However, research showed that MLF1 plays an antiviral role in white spot syndrome virus infection (Feng et al., 2017), so the role of MLF1 in IHNV infection requires in-depth analysis. ARMC5 is a cytosolic protein that was first identified as a tumor suppressor protein in 2013 (Assie et al., 2013), and knockout of the *armc5* gene in mice led to compromised T cell differentiation and proliferation that resulted in a compromised T cell immune response (Hu et al., 2017). PIK3R1 was also downregulated in nervous necrosis virus-infected sea bream larvae (Peruzza et al., 2021). During J-IHNV infection, the *armc5* and *pik3r1* genes were down-regulated compared with U-IHNV infection, and this may suggest that the virulent virus has a stronger inhibitory effect on these genes.

## 5. Conclusion

In this study, a transcriptional analysis of U and J genogroup IHNV-infected RTG-2 cells was performed to explore the molecular mechanisms responsible for the pathogenic differences between U and J IHNV infection. The DEGs induced by IHNV infection were identified and analyzed, and were related to the immune response, signal transduction, and diseases. This report not only provides a comprehensive transcriptional analysis, but also identifies possible mechanisms associated with the differential virulence of U and J genogroup IHNV. Our findings may shed new light on strategies for the prevention of IHNV.

## Data availability statement

The datasets presented in this study can be found in online repositories. The names of the repository/repositories and accession number(s) can be found at: https://www.ncbi.nlm.nih.gov/, PRJNA895736.

## Author contributions

J-ZZ: experiment design, data analysis, and manuscript writing. L-MX: English improvement. G-MR: data curation. Y-ZS: RT-qPCR validation. QL: statistical analysis. C-BT and T-YL: conceptualization, and review and editing. All authors contributed to the article and approved the submitted version.

## Funding

This study was supported by the National Key Research and Development Program of China [2019YFE0115500], the Central Public-interest Scientific Institution Basal Research Fund, Chinese Academy of Fishery Sciences [HSY202204M and 2020TD43], the National Natural Science Foundation of China [32202988], and the Key Research and Development Program of Heilongjiang Province [JD22A017].

## Conflict of interest

The authors declare that the research was conducted in the absence of any commercial or financial relationships that could be construed as a potential conflict of interest.

## Publisher’s note

All claims expressed in this article are solely those of the authors and do not necessarily represent those of their affiliated organizations, or those of the publisher, the editors and the reviewers. Any product that may be evaluated in this article, or claim that may be made by its manufacturer, is not guaranteed or endorsed by the publisher.

## Footnotes

^1^genoscope.cns.fr/trout/data/
